# Investigating the Efficacy of Established Chemical Wood Modifications on Large-Diameter Pine: Durability Against Basidiomycetes

**DOI:** 10.3390/ma18132985

**Published:** 2025-06-24

**Authors:** Lucy S. Martin, Hannes Stolze, Christoph Hötte, Holger Militz

**Affiliations:** Wood Biology and Wood Products, Faculty of Forest Sciences, University of Göttingen, Büsgenweg 4, 37077 Göttingen, Germany; lucy.martin@uni-goettingen.de (L.S.M.); hannes.stolze@uni-goettingen.de (H.S.); christoph.hoette@uni-goettingen.de (C.H.)

**Keywords:** large-diameter pine, SorCA, acetylation, furfurylation, DMDHEU, fungal durability, EN 113-2

## Abstract

In Germany, *Pinus sylvestris* is a dominant tree species, and many trees with large diameters are not utilised due to difficulties with processing. However, older pines have larger volumes of sapwood, and boards with a high sapwood content can be produced. The durability of boards from large-diameter (>50 cm) *P. sylvestris* trees, treated with furfurylation, acetylation, DMDHEU (1.3-dimethylol-4.5-dihydroxyethyleneurea), and SorCA (Sorbitol/Citric Acid), was assessed. The samples were taken from different sections along the longitudinal axis and the cross-section. The durability was tested against *Coniophora puteana*, *Rhodonia placenta*, and *Trametes versicolor*, according to the EN 113-2 standard. All treatments had a median mass loss < 5%, so classed as “highly durable” (Durability Class 1) against all fungi. DMDHEU had a large deviation in mass loss against *Coniophora puteana* and could potentially be classified as “moderately durable” (Durability Class 3), if based on the mean mass loss. The inner part of the end section had a higher mass loss, indicating that there was poorer retention of the treatment at this location. Overall, chemical modifications on large-diameter pine trees were effective at increasing durability. Utilising large-diameter pine trees can help to make use of regional resources and potentially reduce reliance on imported timber. With favourable mechanical properties and easy-to-treat sapwood, large-diameter *P. sylvestris* trees could be used for commercial treatments.

## 1. Introduction

Wood utilisation has become more desirable in recent years as it is a strong, flexible, aesthetically pleasing material and is a renewable resource if sourced sustainably. Sustainable forestry practises can produce quality timber for construction, furniture, and various other uses with a lower environmental impact than competing materials, such as steel or concrete. However, a disadvantage of wood is its susceptibility to biological degradation, particularly fungal decay [1]. To combat this, many products and treatments have been developed to increase the durability of susceptible wood species. This includes chemical modification, which is a well proven method for increasing properties of wood such as durability, hardness, and dimensional stability, although it can reduce some properties such as brittleness [2]. Of the chemical modifications available, furfurylation, acetylation, DMDHEU, and SorCA (sorbitol and citric acid) are among the most well established [3,4,5]. Each uses vacuum-pressure impregnation followed by curing and drying procedures that are carried out in accordance with the manufacturer. In general, chemical modification aims to alter the wood at a cellular level by either filling the lumen, binding to the cell wall, or chemically binding itself and the cell wall, creating cross-links [6,7]. This creates a strong, durable material that can resist fungal decay by reducing access to available moisture, filling the lumen, and blocking fungal pathways or by inhibiting enzymatic digestion of the lignocellulose [8].

However, a requirement for chemical modification is good penetration and retention of the liquid treatment throughout the entire wooden board. Some species restrict the flow of liquid through their anatomical features, such as aspirated pits in spruce wood [9]. Additionally, the sapwood of a tree is more suitable for processes like chemical modification than heartwood due to its more open pits, which allow for the transportation of moisture [10]. Pine sapwood has attractive properties for industrial processes like modification and is generally regarded as easy to treat (Class 1), according to the EN 350 standard [11], although there can be variability between and within different trees [12,13]. One of the most popular species used in chemical wood modification is *Pinus radiata*, which is often sourced from New Zealand plantations to be treated in Europe. However, the regionally available species *Pinus sylvestris* (Scots pine) can also be utilised for chemical modification, and, when sourced from local, sustainably managed forests, it can mitigate climate impacts by reducing the transportation requirements.

With 2.4 million hectares [14], pine is the tree species with the largest area share in German forests (21.8%) and is particularly prevalent in North–East Germany, where over 60% of forested area is *P. slyvestris* [15]. Boards with a high sapwood content (sapwood boards) can be sawn from large-diameter pine trees (>50 cm diameter at breast height [DBH]), which is not possible with small and medium-sized tree diameters. As the current inventory data from German forestry shows, there are large stocks of large-diameter pine trees, which will continue to increase in the future [16]. As *P. sylvestris* sapwood boards have not yet been produced industrially, there is little knowledge about their modification in practical dimensions. Furthermore, due to the low capacity of existing machinery and infrastructure for large-diameter pine trees in Germany, many harvested trees that are above 50 cm DBH are used in lower-quality products, shipped to Asia for further value creation, or are left in forest stands, as there are only limited incentives for utilisation. The KiefernStolz project in Germany identified sawmills in pine regions that are technically capable of producing sapwood boards from large-dimension pine logs and found a higher modification quality for boards with low resin and heartwood content, which can increase the added value of these low-value pines [16].

The aim of this study was to investigate the modification quality and durability of large-diameter (>50 cm DBH) German *Pinus sylvestris* sapwood boards treated with furfurylation, acetylation, SorCA, or DMDHEU. This was achieved via density profile scans used to visualise the distribution of the chemical treatment and via the European Norm EN 113-2 to assess durability in regard to fungal decay. Further, the cross-section, the longitudinal axis, and the board as a whole were evaluated to detect any differences in the retention of the treatment. With this, we aimed to see if sapwood boards from large-dimension German *P. sylvestris* trees could serve as a potential material for commercial treatments.

## 2. Materials and Methods

### 2.1. Test Material and Treatment

Boards of *Pinus sylvestris* sapwood from different locations in Germany were treated with the following chemical modifications according to the process recommended by the institution that produced the material. The treatments and dimensions of the boards are outlined in Table 1.

A general assessment of the wood quality of the boards was carried out. The wood was visually assessed for any signs of cracking, warping, resin leakage, or discolouration after drying and for other wood quality features such as the occurrence and frequency of knots. Then, 60 boards were treated with DMDHEU, 35 with acetylation, 30 with sorbitol and citric acid, 25 with furfurylation and 10 were left untreated as reference boards.

**Figure 1:**
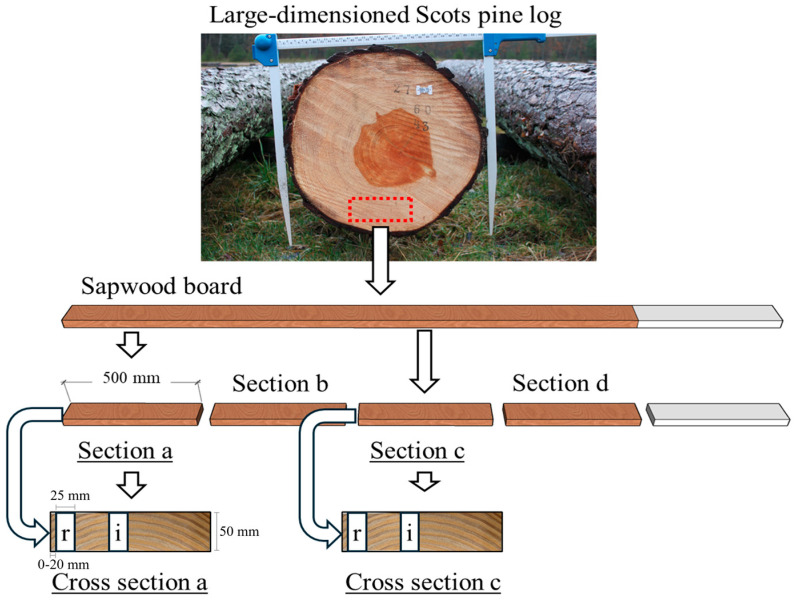
A schematic showing the sectioning of treated *Pinus sylvestris* boards. Fungal test specimens were taken from sections a and c and were separated into those from the inner (i) or edge (r) of the cross-section.

The boards were then sub-divided into 500 mm _(long)_ sections, labelled a–d (Figure 1). Sections a and c were selected for the fungal durability test. From each section, test specimens were cut from either the inner (middle) portion or the edge (0–2 cm from the edge) of the wood. These were labelled ‘i’ and ‘r’, respectively. A mixture of specimens from 28 different boards were tested. All specimens were checked for any deformations or abnormalities prior to the test.

### 2.2. Density Profile

Density profile scans were used to investigate the distribution of chemicals on the cross-section of the modified wood. For this purpose, samples measuring 50 × 35 × 50 (ax.) mm were cut from the centre of the original boards. The measurement was carried out in a kiln-dried state with a DAX 6000 X-ray densitometer (GreCon GmbH, Alfeld, Germany). The samples were oriented in such a way that the radiation direction was parallel to the longitudinal axis of the sample, so that the density profile was displayed in the radial direction, i.e., from one original large board surface to the other. The resolution of the measurement (measurement intervals) was 0.01 mm at a voltage of 33 kV.

### 2.3. Fungal Durability Test

**Figure 2:**
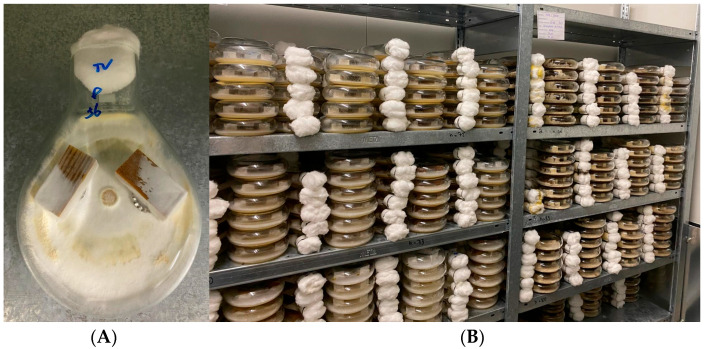
(**A**) Two specimens in a Kolle flask cultured with *Trametes versicolor* on malt agar. (**B**) Flask storage in a culture room at 21 °C and 70% RH for 16 weeks.

The protocol outlined in the European Norm EN 113-2 [17] was followed. Test specimens were cut into 15 × 25 × 50 mm samples. The samples were oven-dried at 103 °C for 48 h and weighed to obtain the oven dry mass before incubation (mL), and then they were conditioned in a climate-controlled room at 20 °C and 65% RH for two weeks. During this time, cultures of the brown-rot fungi *Coniophora puteana* (Cp) and *Rhodonia placenta* (Rp) or the white-rot fungus *Trametes versicolor* (Tv) were grown on malt agar in sterilised, glass Kolle flasks. The samples were then sterilised, and two samples per treatment were placed inside one of the flasks, which was then left in a culture room at 21 °C and 70% RH (Figure 2). Flasks were distributed randomly throughout the culture room to avoid location bias. After 16 weeks, the samples were removed, carefully cleaned of any external fungal mycelium, and weighed to obtain the wet mass after incubation (*m*_2_). They then underwent a stepwise drying procedure in an oven at 50°, 70°, 90°, and 103 °C for 48 h each and were weighed again to obtain the dry mass after incubation (*m*_3_).

Overall, 68 samples per treatment/per fungus were tested in addition to 32 untreated reference *P. sylvestris* samples and 30 untreated beech samples (to act as fungi virulence controls). In total, 204 samples of each treatment were tested, with 96 untreated reference samples and 90 untreated beech samples.

The moisture content (*MC*) and mass loss (*ML*) of each specimen were calculated using Equations (1) and (2), respectively.

(1) $$MC=\frac{m_{2}-m_{3}}{m_{3}}\cdot 100$$

(2) $$ML=\frac{m_{1}-m_{3}}{m_{1}}\cdot 100$$

where *m*_1_ is the oven dry mass before incubation, *m*_2_ is the wet mass after incubation, and *m*_3_ is the oven dry mass after incubation.

The median *ML* was used to evaluate durability classes according to EN 113-2 (Table 2).

## 3. Results

### 3.1. Density Profiles

**Figure 3:**
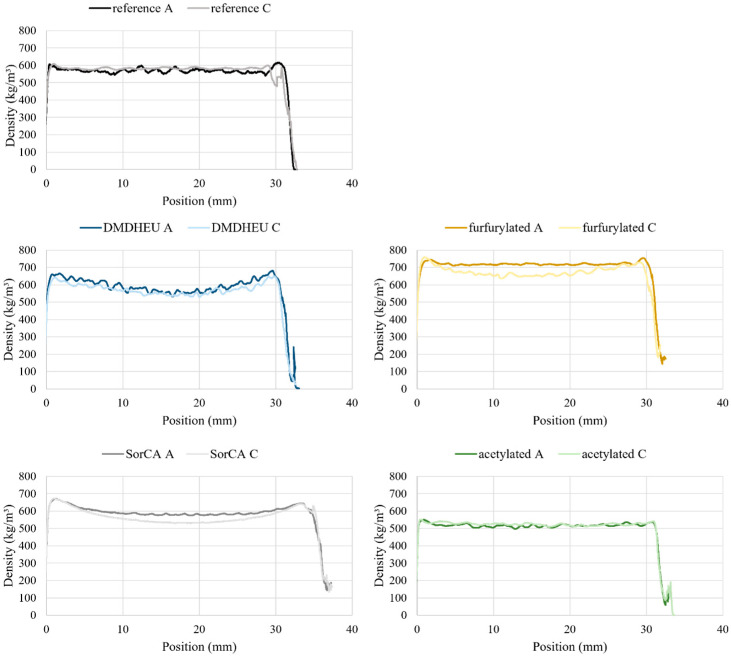
Density profiles of untreated (reference) and modified pine samples (DMDHEU, furfurylated, SorCA, and acetylated) depending on their position in the board.

Chemical gradients between the inner and outside wood were seen with the furfurylated, DMDHEU-treated, and SorCA-treated samples (Figure 3). In the furfurylated and SorCA samples, the density at the ends of the boards was also higher, which indicated that the chemicals migrated in a lateral direction towards the surfaces. There was also migration in the longitudinal direction towards the ends of the boards. Nevertheless, these gradients were not very high and were to be expected due to the migration of chemicals during the drying and curing processes. Table 3 gives the density at the centre of the samples divided by the maximum density and multiplied by 100. This shows the proportion of the sample that has a density equal to its maximum density, i.e., the lower the value, the higher the density gradient is. DMDHEU A and SorCA C had the highest density gradients of 77.71% and 79.10%, respectively.

### 3.2. Fungi Test Results

**Figure 4:**
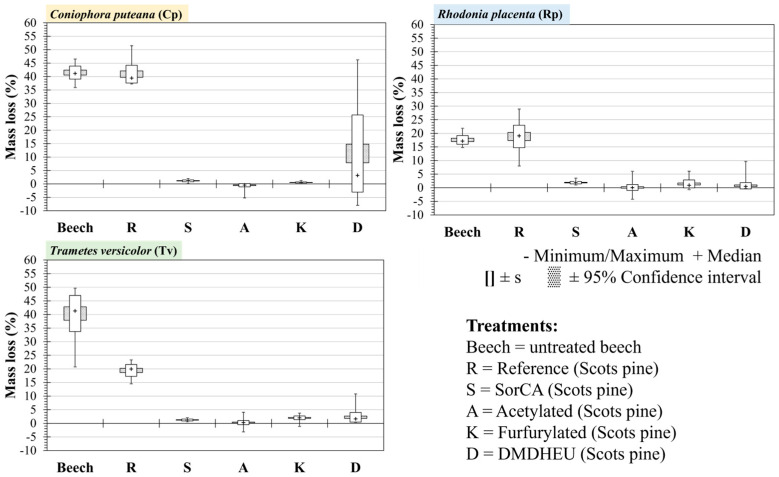
Mass loss of modified Scots pine wood against the brown-rot fungi *Coniophora puteana* and *Rhodonia placenta* and the white-rot fungus *Trametes versicolor*. Median marker (+), 95% confidence interval, and standard deviation(s) are shown.

The median mass loss (ML) of the reference samples (R) and untreated beech reached the recommended minimum of 20% ML for Cp and Tv and reached 19.08% and 17.14%, respectively, for Rp (Figure 4, Table A1). This showed that the fungal strains used were virulent enough to validate the test, according to EN 113-2. Although the pine reference for Rp did not reach 20% median ML, half of the individual specimens had an ML > 19%, and this was considered sufficient as at least one of the fungi/reference combinations had a median ML > 30%. Moisture content was also calculated for all specimens and is presented in Appendix A Table A1, Table A2 and Table A3.

The median ML for all the modified wood against all fungi was below 5%, indicating that they were all highly durable (DC1). However, for the DMDHEU treatment against *Coniophora puteana*, there was a very large standard deviation, and the mean ML was 11.32%, which would be equivalent to DC3 (moderately durable). The reason for this deviation was further explored by separating the samples according to the location that they were taken from on the longitudinal axis and cross-section of the board.

**Figure 5:**
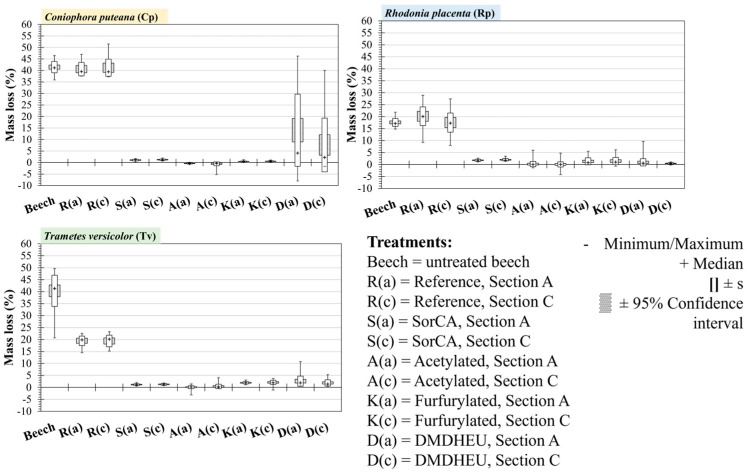
Mass loss of modified Scots pine wood against the brown-rot fungi *Coniophora puteana* and *Rhodonia placenta* and the white-rot fungus *Trametes versicolor*. Median marker (+), 95% confidence interval, and standard deviation(s) are shown. Modification treatments and reference Scots pine were separated if they were sampled from section A or section C.

The samples from the end section (A) of the treated boards did not appear to have any difference in durability compared to the middle section (C) (Figure 5). For DMDHEU against Cp, large variations were still seen for both sections A and C, although section A had a slightly higher median ML of 4.13%, compared to 2.25% (Table A2). However, according to EN 113-2, a median ML < 5% is still considered durable for both.

**Figure 6:**
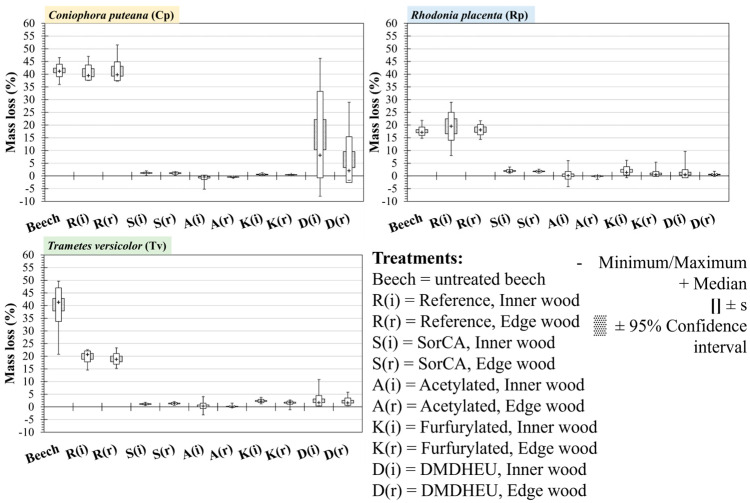
Mass loss of modified Scots pine wood against the brown-rot fungi *Coniophora puteana* and *Rhodonia placenta* and the white-rot fungus *Trametes versicolor*. Median marker (+), 95% confidence interval, and standard deviation(s) are shown. Modification treatments and reference Scots pine samples are separated if they were sampled from the inner (middle) portion (i) or the edge (0–2 cm from the edge) of the wood (r).

A difference between the inner (i) and edge (r) sections was not evident in almost all of the treatments and fungi species that were tested (Figure 6). Only against Cp did DMDHEU exhibit a higher mass loss of 8.13% in the inner section, compared to 2.05% at the edge (Table A3).

**Figure 7:**
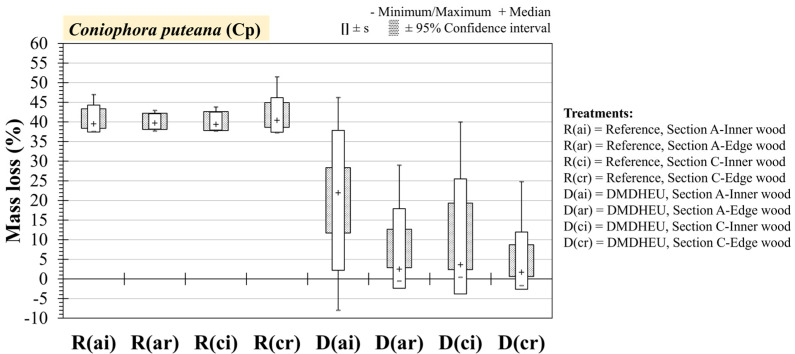
Mass loss (ML) of Scots pine wood modified with DMDHEU against the brown-rot fungus *Coniophora puteana* (Cp). Median marker (+), 95% confidence interval, and standard deviation(s) are shown. DMDHEU-treated samples and the untreated Scots pine reference samples are separated depending on if they were sampled from the inner (middle) portion (i) or the edge (0–2 cm from the edge) of the wood (r) and from section A (a) or section C (c) along the longitudinal axis.

To further investigate the variation in the mass loss of DMDHEU against *Coniophora puteana*, samples from the inner/edge (i/r) and sections A/C are plotted in Figure 7. Although all groups still presented an unusually large deviation, the inner part of the end-most section (Ai) had a much higher median ML of 20.25% (Table A4) than the other sections. According to Table 2, this is considered to be DC4 ‘slightly durable’, whereas the inner part of section C (Ci) and the edge parts of section A (Ar) and C (Cr) all had a median mass loss < 5%, classing them as DC1.

Despite the large difference in the median value between the different groups of DMDHEU-treated wood, following a Kruskal–Wallis test, there were no significant differences found (χ^2^ = 67, *p* = 0.477). Nevertheless, further investigation using a Dunn post hoc test found the biggest difference to be between group D(ai) and D(cr), although this was still not significant (*p* = 0.0378) with the Bonferroni correction applied to the alpha value (α = 0.025).

## 4. Discussion

Along the longitudinal axis of a treated board, the section closest to the end grain can sometimes have a higher concentration of the chemical compared to the middle sections because of migration during curing [4]. This may leave the middle of the board with a low retention of the treatment and more susceptible to decay. The migration of the chemical treatment can also occur towards the edge of the board during curing and drying [18,19]. This is because much of the water solvent is removed during this process, which can carry unfixed chemicals to the edges. This may happen particularly at the beginning of the curing process as temperatures may still be below the fixation temperature of the treatment. Consequently, the inner part of the cross-section can also experience a lower level of treatment and therefore be more vulnerable to decay. Since we used pine sapwood in all cases, we can assume that the samples were completely saturated during impregnation. So, rather than being an effect of the insufficient penetration of the liquid treatment, it is likely that during curing, some of the chemicals were transported towards the surfaces with escaping water. This would result in the inner section, particularly at the end grain of the board, retaining a lower amount of the treatment and therefore experiencing higher levels of decay.

DMDHEU exhibited the largest gradient in the density profile from the inner section to the edge of cross-section, which may explain the difference observed in mass loss between these sections. Nevertheless, DMDHEU has been shown to have high durability against soft-rot and basidiomycete fungi in both rapid laboratory (EN 113, ENV807) and long-term field (EN252) tests, particularly with Scots pine sapwood [4,20]. Variations in results may arise between studies in such monoculture tests, as identified by [20], due to several factors, including the curing and fixation of the treatment and differing fungal strains and laboratory set ups. Given that the other treatments did not experience the same large deviations, the variation in DMDHEU seen in this study will be further investigated. In other investigations of laboratory-scale samples treated with DMDHEU, one of the major modes of action identified w as the distribution and fixation of the chemicals, and this directly affected durability against fungal decay [21]. Drying and curing regimes after treatment can be adapted to mitigate migration. Drying, pre-drying, and curing in dry conditions can lead to increased migration of the chemical away from the centre of the plank, so maintaining a high moisture content of the wood can reduce this [22]. However, the increase in moisture can affect chemical fixation and increase emissions of formaldehyde, therefore curing in a super-heated steam atmosphere was found to result in a good fixation and distribution of DMDHEU [23].

As for the other treatments, SorCA and, to a lesser extent, furfurylation also showed a gradient in the density profile, but there were no apparent differences in durability in the two sections tested. Highly durable treatments such as acetylation showed small amounts of negative mass loss. This is likely related to unremoved fungal mycelium, which may still be present inside of the sample, or from a slight uptake of moisture from the air as the dry samples were being weighed.

In general, large-diameter *P. sylvestris* was easy to treat, although there can be issues with the migration of the chemical treatment during curing. An undesirable trait for treating pine boards is a high heartwood content containing resin-rich areas. This makes impregnation treatments more difficult and results in an ununiform distribution of the modification. Therefore, heartwood content should be reduced as much as possible, and for this reason, *Pinus radiata* is commonly used for commercial modification (such as by Kebony and Accsys) due to the low proportion of heartwood [2]. This could also be achieved by sawing sapwood boards from older, large-diameter trees. Utilising large-diameter *Pinus sylvestris* trees from regional forests could be sustainable and reduce reliance on plantation-grown *Pinus radiata* from New Zealand. This would be beneficial in mitigating climate impacts that are associated with the growth and transportation of radiata pine. Additionally, *P. sylvestris* has a high density, giving it favourable mechanical properties, and is able to tolerate harsh climatic conditions, such as drought and nutrient-poor soils [24]. This, along with low-resin, easy-to-treat sapwood, can make for an ideal choice in commercial treatments, and if sapwood boards from large-dimension German *P. sylvestris* trees are used, process optimisation is an important next step.

## 5. Conclusions

We were able to treat large-diameter *Pinus sylvestris* from Germany using four well-known chemical wood modifications. All treatments improved durability against *Coniophora puteana, Rhodonia placenta*, and *Trametes versicolor*. Furfurylation, acetylation, and SorCA were considered ‘highly durable’ (Durability Class 1) and did not vary based on whether the sample was taken from the section along the longitudinal axis or the inner/outer part of the cross-section. DMDHEU had a large standard deviation, which was most prominent at the inner part of the end-most section of the board, indicating poor retention of the treatment at this location. However, the median mass loss for the DMDHEU treatment was <5%, which is still classified as Durability Class 1. The application of large-diameter *Pinus sylvestris* for wood modification could increase the utilisation of regionally grown German timber, and given these promising initial results, further testing, for example, outdoor field trials, can follow.

## Figures and Tables

**Table 1:** Chemical modifications applied to large-diameter *Pinus sylvestris* trees, treated in accordance with the institution that produced the material.

Abbreviation	Modification	Institution	Board Dimensions [mm]	Concentration/ WPG (%)
S	Sorbitol/citric acid (SorCA)	University of Göttingen, Göttingen, Germany	35 × 85 × 1700 _(long)_	30/19.5
A	Acetylation	Accsys, Arnhem, The Netherlands	32 × 75 × 2500 _(long)_ or 32 × 100 × 2500 _(long)_	-/22.8
K	Furfurylation	Kebony, Skien, Norway	32 × 75 × 2500 _(long)_ or 32 × 100 × 2500 _(long)_	-/57.6
D	DMDHEU	University of Göttingen, Göttingen, Germany	30 × 145 × 1400 _(long)_	25/32.3
R	None (reference)	University of Göttingen, Göttingen, Germany	/	/
B	None (untreated beech)	University of Göttingen, Göttingen, Germany	/	/

**Table 2:** Durability classes according to EN 113-2 (2020).

Durability Class	Description	Median Mass Loss (%)
DC1	Highly durable	≤5
DC2	Durable	>5 to ≤10
DC3	Moderately durable	>10 to ≤15
DC4	Slightly durable	>15 to ≤30
DC5	Not durable	>30

**Table 3:** Proportion of the sample area with a density equal to its maximum density.

Treatment		%
DMDHEU	A	77.71
	C	82.43
Acetylated	A	93.27
	C	93.30
Furfurylated	A	95.35
	C	85.11
SorCA	A	86.12
	C	79.10
Reference	A	93.34
	C	95.73

## Data Availability

The raw data supporting the conclusions of this article will be made available by the authors upon request.

## Abbreviations

The following abbreviations are used in this manuscript:

ax.	Axial
Cp	*Coniophora puteana*
DBH	Diameter at breast height
DMDHEU	1.3-dimethylol-4.5-dihydroxyethyleneurea
long.	Longitudinal
MC	Moisture content
ML	Mass loss
RH	Relative humidity
Rp	*Rhodonia placenta*
SorCA	Sorbitol and citric acid
Tv	*Trametes versicolor*

## Appendix A

**Table A1:** Mass loss (ML) and moisture content (MC) of modified Scots pine wood against the brown rot fungi *Coniophora puteana* (Cp) and *Rhodonia placenta* (Rp) and the white rot fungus *Trametes versicolor* (Tv). Durability class (DC) is given in accordance with EN 113-2 (2021) based on the median mass loss of all the specimens. The mean DC is additionally given as the average durability class received by each specimen. See Table 1 for treatment abbreviations.

Fungus	Treatment	Mean MC (%)	Median ML (%)	Mean ML (%)	SD ML (%)	DC	Mean DC
Cp	S	54.62	1.18	1.14	0.37	1	1.00
	A	50.92	−0.42	−0.49	0.61	1	1.00
	K	50.20	0.52	0.53	0.22	1	1.00
	D	70.55	3.15	11.32	14.27	1	2.22
	R	73.34	39.44	40.90	3.26	5	5.00
	B	69.05	41.15	41.47	2.40	5	5.00
Rp	S	29.61	1.84	1.88	0.47	1	1.00
	A	17.86	0.05	0.11	1.09	1	1.01
	K	31.74	0.92	1.40	1.47	1	1.06
	D	41.50	0.49	0.73	1.15	1	1.01
	R	91.14	19.08	18.85	4.05	4	3.81
	B	92.31	17.14	17.60	1.60	4	3.97
Tv	S	52.07	1.20	1.24	0.35	1	1.00
	A	23.83	0.23	0.27	0.78	1	1.00
	K	55.72	1.98	2.01	0.69	1	1.00
	D	79.63	1.62	2.24	1.77	1	1.07
	R	57.08	19.95	19.45	2.13	4	3.97
	B	60.23	41.32	40.36	6.51	5	4.93

**Table A2:** Mass loss (ML) and moisture content (MC) of modified Scots pine wood against the brown rot fungi *Coniophora puteana* (Cp) and *Rhodonia placenta* (Rp) and the white rot fungus *Trametes versicolor* (Tv). Samples are separated based on the section they were taken from along the longitudinal axis: section (A) or (C). Durability class (DC) is given in accordance with EN 113-2 (2021) based on the median mass loss of all the specimens. The mean DC is additionally given as the average durability class received by each specimen. See Table 1 for treatment abbreviations.

Fungus	Treatment	Mean MC (%)	Median ML (%)	Mean ML (%)	SD ML (%)	DC	Mean DC
Cp	S(A)	54.69	1.14	1.07	0.39	1	1.00
	S(C)	54.53	1.20	1.23	0.31	1	1.00
	A(A)	50.88	−0.44	−0.41	0.21	1	1.00
	A(C)	50.96	−0.40	−0.57	0.85	1	1.00
	K(A)	50.79	0.49	0.49	0.21	1	1.00
	K(C)	49.54	0.54	0.56	0.22	1	1.00
	D(A)	69.67	4.13	14.06	15.49	1	2.56
	D(C)	71.73	2.25	7.64	11.48	1	1.76
	R(A)	72.55	39.52	40.59	2.83	5	5.00
	R(C)	74.14	39.43	41.20	3.62	5	5.00
	B	69.05	41.15	41.47	2.40	5	5.00
Rp	S(A)	29.07	1.74	1.78	0.42	1	1.00
	S(C)	30.26	1.86	2.00	0.49	1	1.00
	A(A)	17.35	0.06	0.19	1.10	1	1.03
	A(C)	18.32	0.03	0.03	1.07	1	1.00
	K(A)	31.77	0.82	1.33	1.45	1	1.06
	K(C)	31.71	1.03	1.48	1.48	1	1.06
	D(A)	42.61	0.57	0.92	1.49	1	1.03
	D(C)	40.09	0.46	0.50	0.28	1	1.00
	R(A)	90.59	20.04	20.16	3.79	4	3.88
	R(C)	91.69	17.29	17.54	3.87	4	3.75
	B	92.31	17.14	17.60	1.60	4	3.97
Tv	S(A)	50.62	1.17	1.19	0.34	1	1.00
	S(C)	53.71	1.28	1.29	0.35	1	1.00
	A(A)	24.14	0.19	0.14	0.61	1	1.00
	A(C)	23.46	0.25	0.42	0.91	1	1.00
	K(A)	56.79	1.97	1.94	0.48	1	1.00
	K(C)	54.44	1.99	2.09	0.86	1	1.00
	D(A)	82.31	1.91	2.60	2.09	1	1.12
	D(C)	76.96	1.37	1.88	1.27	1	1.03
	R(A)	57.93	19.86	19.49	1.90	4	3.94
	R(C)	56.23	20.15	19.40	2.33	4	4.00
	B	60.23	41.32	40.36	6.51	5	4.93

**Table A3:** Mass loss (ML) and moisture content (MC) of modified Scots pine wood against the brown rot fungi *Coniophora puteana* (Cp) and *Rhodonia placenta* (Rp) and the white rot fungus *Trametes versicolor* (Tv). Samples are separated based on the location of the cross section they were taken from the inner section (i); or the edge (r). Durability class (DC) is given in accordance with EN 113-2 (2021) based on the median mass loss of all the specimens. The mean DC is additionally given as the average durability class received by each specimen. See Table 1 for treatment abbreviations.

Fungus	Treatment	Mean MC (%)	Median ML (%)	Mean ML (%)	SD ML (%)	DC	Mean DC
Cp	S(i)	51.76	1.17	1.17	0.28	1	1.00
	S(r)	57.48	1.23	1.12	0.44	1	1.00
	A(i)	52.75	−0.40	−0.54	0.84	1	1.00
	A(r)	49.09	−0.44	−0.43	0.18	1	1.00
	K(i)	50.46	0.54	0.58	0.27	1	1.00
	K(r)	49.94	0.50	0.48	0.14	1	1.00
	D(i)	73.23	8.13	16.24	16.75	2	2.76
	D(r)	67.87	2.05	6.40	8.87	1	1.68
	R(i)	73.88	39.44	40.62	2.90	5	5.00
	R(r)	72.81	39.76	41.17	3.57	5	5.00
	B	69.05	41.15	41.47	2.40	5	5.00
Rp	S(i)	29.34	1.85	1.95	0.57	1	1.00
	S(r)	29.89	1.83	1.81	0.32	1	1.00
	A(i)	17.69	0.11	0.27	1.49	1	1.03
	A(r)	18.04	0.00	−0.06	0.31	1	1.00
	K(i)	32.68	1.38	2.00	1.64	1	1.09
	K(r)	30.81	0.51	0.80	0.94	1	1.03
	D(i)	42.31	0.53	0.90	1.57	1	1.03
	D(r)	40.69	0.48	0.57	0.36	1	1.00
	R(i)	92.44	19.57	19.51	5.30	4	3.69
	R(r)	89.84	18.11	18.20	1.96	4	3.94
	B	92.31	17.14	17.60	1.60	4	3.97
Tv	S(i)	48.31	1.12	1.13	0.28	1	1.00
	S(r)	55.83	1.44	1.35	0.38	1	1.00
	A(i)	22.07	0.31	0.40	1.05	1	1.00
	A(r)	25.58	0.08	0.13	0.28	1	1.00
	K(i)	56.65	2.23	2.35	0.59	1	1.00
	K(r)	54.78	1.78	1.66	0.60	1	1.00
	D(i)	80.62	1.66	2.42	2.03	1	1.09
	D(r)	78.65	1.59	2.06	1.44	1	1.06
	R(i)	57.08	20.68	19.95	2.10	4	3.94
	R(r)	57.08	18.81	18.95	2.04	4	4.00
	B	60.23	41.32	40.36	6.51	5	4.93

**Table A4:** Mass loss (ML) and moisture content (MC) of Scots pine wood modified with DMDHEU against the brown rot fungus *Coniophora puteana* (Cp). Samples are separated based on the section they were taken from along the longitudinal axis, section (A) or (C), and the location they were taken from, i.e., the cross-section of the wood; the inner section (i), or the edge (r). Durability class (DC) is given in accordance with EN 113-2 (2021) based on the median mass loss of all the specimens. The mean DC is additionally given as the average durability class received by each specimen.

Fungus	Treatment	Mean MC (%)	Median ML (%)	Mean ML (%)	SD ML (%)	DC	Mean DC
Cp	R(Ai)	74.01	39.52	40.86	3.27	5	5.00
	R(Ar)	70.12	39.70	40.15	1.79	5	5.00
	R(Ci)	73.65	39.38	40.21	2.10	5	5.00
	R(Cr)	74.43	40.45	41.79	4.17	5	5.00
	D(Ai)	70.19	20.25	19.26	17.49	4	3.16
	D(Ar)	69.18	2.49	7.77	9.86	1	1.84
	D(Ci)	77.64	3.63	10.83	14.14	1	2.07
	D(Cr)	66.22	1.73	4.67	7.05	1	1.47
